# Field-friendly anti-PGL-I serosurvey in children to monitor *Mycobacterium leprae* transmission in Bihar, India

**DOI:** 10.3389/fmed.2023.1260375

**Published:** 2023-09-27

**Authors:** Louise Pierneef, Paritosh Malaviya, Anouk van Hooij, Shyam Sundar, Abhishek Kumar Singh, Rajiv Kumar, Danielle de Jong, Maaike Meuldijk, Awnish Kumar, Zijie Zhou, Kristien Cloots, Paul Corstjens, Epco Hasker, Annemieke Geluk

**Affiliations:** ^1^Department of Infectious Diseases, Leiden University Medical Center, Leiden, Netherlands; ^2^Department of Medicine, Institute of Medical Sciences, Banaras Hindu University, Varanasi, India; ^3^Centre of Experimental Medicine and Surgery, Institute of Medical Sciences, Banaras Hindu University, Varanasi, India; ^4^Department of Cell and Chemical Biology, Leiden University Medical Center, Leiden, Netherlands; ^5^Department of Public Health, Institute of Tropical Medicine, Antwerp, Belgium

**Keywords:** children, leprosy, anti-*M. leprae* PGL-I antibodies, infection, diagnostics, serosurvey, transmission, upconversion

## Abstract

**Background:**

It has been amply described that levels of IgM antibodies against *Mycobacterium leprae* (*M. leprae*) phenolic glycolipid I (PGL-I) correlate strongly with the bacterial load in an infected individual. These findings have generated the concept of using seropositivity for antibodies against *M. leprae* PGL-I as an indicator of the proportion of the population that has been infected. Although anti-PGL-I IgM levels provide information on whether an individual has ever been infected, their presence cannot discriminate between recent and past infections. Since infection in (young) children by definition indicates recent transmission, we piloted the feasibility of assessment of anti-PGL-I IgM seroprevalence among children in a leprosy endemic area in India as a proxy for recent *M. leprae* transmission.

**Material and methods:**

A serosurvey for anti-PGL-I IgM antibodies among children in highly leprosy endemic villages in Bihar, India, was performed, applying the quantitative anti-PGL-I UCP-LFA cassette combined with low-invasive, small-volume fingerstick blood (FSB).

**Results:**

Local staff obtained FSB of 1,857 children (age 3–11 years) living in 12 leprosy endemic villages in Bihar; of these, 215 children (11.58%) were seropositive for anti-PGL-I IgM.

**Conclusion:**

The anti-PGL-I seroprevalence level of 11.58% among children corresponds with the seroprevalence levels described in studies in other leprosy endemic areas over the past decades where no prophylactic interventions have taken place. The anti-PGL-I UCP-LFA was found to be a low-complexity tool that could be practically combined with serosurveys and was well-accepted by both healthcare staff and the population. On route to leprosy elimination, quantitative anti-PGL-I serology in young children holds promise as a strategy to monitor recent *M. leprae* transmission in an area.

## Introduction

Leprosy, caused by *Mycobacterium leprae (M. leprae)* or *M. lepromatosis*, is a debilitating neglected tropical disease (NTD) still predominantly forming a health threat for poor and marginalized populations from over 120 countries (1–3). It is a chronic infectious disease that can cause long-term nerve damage and often results in both physical and social disabilities (4, 5). *M. leprae* is believed to be transmitted via aerosol droplets from the respiratory system, during repeated and close contact with untreated patients (6). Only approximately 5% of persons infected with *M. leprae* develop disease symptoms (4). However, it is assumed that infected, asymptomatic individuals carrying sufficient amounts of the mycobacterium contribute to its transmission (7, 8).

Multidrug therapy (MDT) effectively kills *M. leprae* and provides an effective cure if treatment is initiated timely. Following MDT's introduction in 1981, leprosy prevalence has significantly dropped. Yet, transmission of *M. leprae* remains, reflected by over 9,000 new child cases detected in 2022 worldwide (9). It is also believed that large numbers go undetected as a result of the drop in leprosy-focused healthcare following the declaration of leprosy elimination on the global level (10).

The WHO's Global Leprosy Strategy 2021–2030 aims to significantly reduce the number of new cases with grade 2 disability and new child cases by focusing on early detection of disease and interruption of transmission. To achieve the latter, it is vital to identify and treat sources of infection, e.g., multibacillary (MB) cases with high bacillary loads that are highly likely to transmit bacteria (11, 12). Detecting *M. leprae*-infected individuals lacking clinical symptoms who can transmit the bacterium or develop leprosy themselves remains a major challenge for dedicated control programs. Monitoring leprosy elimination is currently conducted by evaluating the proportion of new child cases (below age 15) among the total number of new cases detected (9, 13, 14). As only a small percentage of *M. leprae*-infected individuals progress to disease and it can take many years before symptoms of leprosy manifest (15), using new cases to monitor elimination does not provide sufficiently accurate and up-to-date information with respect to (elimination of) transmission.

Household contacts of MB leprosy cases have been reported to be most vulnerable to contracting disease (16, 17). Levels of IgM antibodies against phenolic glycolipid I (PGL-I), a specific cell wall component of *M. leprae*, correlate strongly with the bacterial index (BI) of *M. leprae*-infected individuals (8, 18, 19). Moreover, based on a literature review covering reports on serology for leprosy from 1987 to 2020 worldwide, we showed that quantitative anti-PGL-I serology in young children as a measure for *M. leprae* infection provides the potential for assessing recent transmission rates in a community (20, 21). These findings, on top of the observation that MB cases are more likely to transmit bacteria, provide a rationale for identifying seropositive individuals to study transmission. Although the presence of antibodies cannot discriminate between recent and past infection, infection in young children by definition indicates recent transmission. Therefore, the assessment of seropositivity in children is recommended by the WHO “Task Force on *definitions, criteria and indicators for interruption of transmission and elimination of leprosy*” as an indicator to monitor elimination (9) and offers a tool to study the effects of interventions including post-exposure prophylaxis (PEP).

Over the past years, we have developed a robust, user-friendly test that detects anti-PGL-I IgM antibodies using the unique upconverting reporter particle (UCP) technology in a low-cost lateral flow-based assay (LFA) format [(18, 22–26) and Pierneef et al. (*manuscript in preparation*)]. The anti-PGL-I UCP-LFA offers a sensitive, low-complexity rapid test to quantitatively determine anti-PGL-I IgM levels in capillary and venous blood (24, 27).

In India, the new leprosy child case detection rate was approximately 10 per 1,000,000 child population in 2020 (28). Of the new cases of all ages registered in India, 15 to 20% (16,000–20,000 individuals each year) are located in Bihar (29), a socio-economically poor state in the eastern part of the country with an estimated population of over 100 million people (30). In this study, we report for the first time the application of the fully integrated anti-PGL-I UCP-LFA cassette in a larger serosurvey using fingerstick blood (FSB). We aimed to assess the use of the anti-PGL-I UCP-LFA as a tool for measuring *M. leprae* infection among children as a proxy for transmission in Bihar, India.

## Methods

### Study participants

Study participants were consenting individuals living in a Health and Demographic Surveillance System (HDSS) site in Muzaffarpur district, Bihar, India.

#### Children cohort

As part of the Tropical Medicine Research Center (TMRC) study on leishmaniasis in the already-existing Muzaffarpur HDSS, a door-to-door screening was performed by the staff of the Banaras Hindu University (BHU; Varanasi, India) (31, 32). The screening started in August 2020 and was completed in January 2021. FSB from children (*n* = 1,857; 987 male/870 female) between 3 and 11 years of age living in Bihar was collected (Table 1). The inclusion criteria were all children without any signs of leprosy or other diseases residing permanently in one of the following villages in the old HDSS area (32): Singar Phulkahan, Madhopur Chhapra, Godai Phulkahan, Godai Jamal, Vishwanathpur, Raksha North, Raksha North Chauk, Raksha South West, Raksha South, Raksha Deah, Nariyar Nawada, and Arizpur Kothi. Excluded were children below 2 years of age. BCG status was not recorded in this study but has been above 84% and almost uniform across India since the time of the study (2020) (33).

**Table 1:** Age and gender of the 1,857 children per village of residence.

**Village**	**Village name**	**# Children**	**Age mean (range)**	**Gender (% female)**
A	Singar Phulkahan	114	7 (3–9)	43.86
B	Madhopur Chhapra	126	7 (5–9)	45.24
C	Godai Phulkahan	216	7 (5–9)	50.46
D	Godai Jamal	110	7 (5–9)	45.45
E	Vishwanathpur	89	7 (5–9)	49.44
F	Raksha North	164	7 (5–11)	49.39
G	Raksha North Chauk	204	7 (5–9)	48.04
H	Raksha South West	97	7 (5–9)	50.52
I	Raksha South	411	7 (5–9)	47.69
J	Raksha Deah	250	7 (3–9)	40.80
K	Nariyar Nawada	70	7 (5–9)	48.57
L	Arizpur Kothi	6	7 (5–9)	0
	Total	1,857	7 (3–9)	46.85

The number (#) of children, mean age (range), and percentage of females per village of residence are provided.

#### Leishmaniasis

In addition, the following biobank samples collected during the TMRC study in Bihar on leishmaniasis were included.

Serum samples from leishmaniasis patients from the same area were collected, including visceral leishmaniasis (VL; age 12–52; *n* = 20) and post-kala-azar dermal leishmaniasis (PKDL; age 12–62; *n* = 20). VL patients were rK39 antibody/splenic aspirate positive and displayed clinical symptoms of active VL. PKDL patients were rK39 antibody or skin slit smear/PCR positive and had a history of VL (34).

#### Asymptomatic individuals

Serum samples from asymptomatic individuals (ASY; age 10–70) were collected. ASY were individuals without any clinical symptoms of leishmaniasis, who were seropositive for rK39 antibodies in the Direct Agglutination Test (DAT; cutoff titer for positivity ≥1:1,600) (34).

#### Endemic controls

Serum samples from endemic controls (ECs; age 8–50; *n* = 20) were collected as part of the NIH-TMRC project. ECs were individuals who tested seronegative for rK39 antibodies/DAT (34).

### Fingerstick blood collection

FSB was collected using disposable 20 μl Minivette^®^ collection tubes (Heparin coated; Sarstedt) and directly mixed with 980 μl high salt finger stick (HSFS) buffer: 100 mM Tris pH 8.0, 270 mM NaCl, 1% (v/v) Triton X-100, and 1% (w/v) BSA. FSB was applied to the anti-PGL-I UCP-LFA cassette on the spot immediately after collection.

### Serum collection

For samples that were used as positive and negative controls throughout the study, venous blood samples were collected, in 4 ml plain BD vacutainer serum tubes (BD, Franklin Lakes, NJ, USA). Tubes were centrifuged at an RCF of 500 xg for 10 min and sera were subsequently aliquoted and frozen (−80°C) until use.

### Anti-PGL-I UCP-LFA cassette

**Figure 1:**
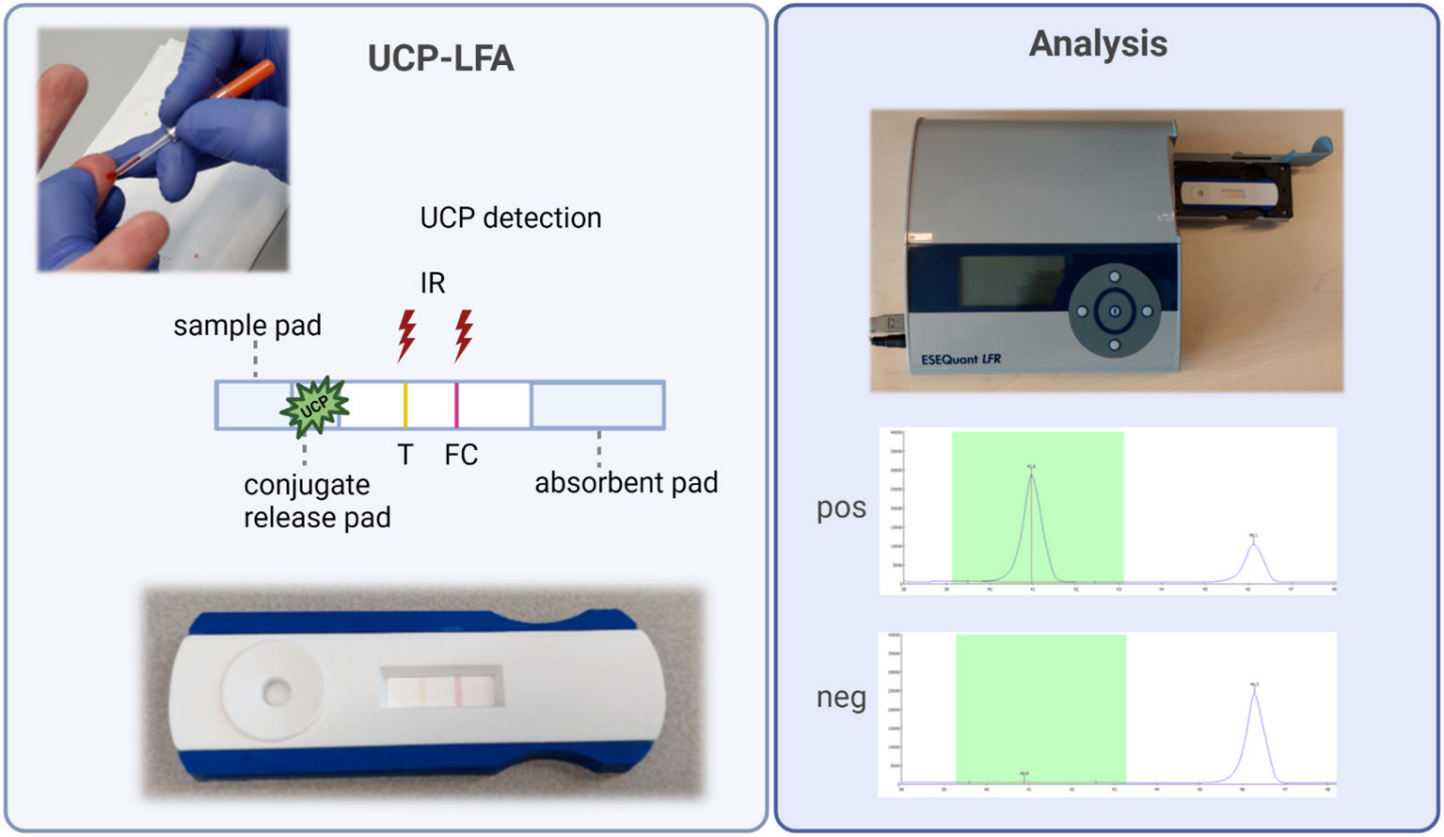
Fully integrated anti-PGL-I UCP-LFA cassette and analysis. **Left:** FSB is collected with a minivette (20 μl). To initiate LF, a diluted FSB sample is added to the cassette onto the sample pad. Hydration of the anti-IgM UCP-conjugate will allow the binding of anti-PGL-I IgM antibodies from the sample to the conjugate and sequential binding to the T line with synthetic PGL-I. The FC line can bind UCP conjugate not bound to the T line. Color-coded T and FC lines visible by the eye disappear upon flow. **Right:** UCP signals are detected with a reader upon excitation with 980 IR light generating 540 nm emission. Results for positive and negative samples are shown. Results are calculated as R-value, with the T signal divided by the FC signal. This figure was created with BioRender.com. FC, flow control line; FSB, fingerstick blood; IgM, immunoglobulin M; IR, infrared excitation; PGL-I, phenolic glycolipid I; R, ratio value, result of the UCP-LFA; T, test line; UCP-LFA, upconverting reporter particle lateral flow assay.

Fully integrated and individually packaged UCP-LFA cassettes for the detection of human anti-PGL-I IgM antibodies were produced by MaximBio (Rockville, MD, USA; schematic overview shown in Figure 1). The air-tight pouches with test cassettes contained silica dry packs allowing extended shelf life and protection against humidity. The Test (T) line on the LF strip comprised 100 ng of synthetic PGL-I, phenolic trisaccharide functionalized with a hexanoic acid linker for conjugation to BSA [NPT1-H-BSA; Leiden, the Netherlands (35)]. The Flow-Control (FC) line comprised 100 ng rabbit anti-goat IgG [G4018; Sigma-Aldrich, Inc., St. Louis, MO, USA). Goat IgG specific for anti-human IgM (I0759; Sigma-Aldrich, Inc., St. Louis, MO, USA] was conjugated to polyacrylic acid functionalized UCPs [200 nm, NaYF_4_:Yb^3+^, Er ^3+^; Intelligent Material Solutions Inc. (IMS); Princeton, NJ, USA MS] according to previously described protocols at a concentration of 50 μg antibody per mg UCP (23). Stock solutions were kept at 4°C until use. To dry the UCPs onto the glass fiber conjugate-release pad, the material was diluted in a buffer containing 100 mM Tris pH 8.0, 270 mM NaCl, 10% (w/v) sucrose, 1% (w/v) BSA, 0.5% Tween-20, and striped at a density of 100 ng/mm. Components were mounted on plastic backing cards which were cut into LF strips of 4.8 mm width by 6 cm length, added to an appropriate cassette, and individually sealed in a pouch together with a silica dry pack.

### UCP-LFA

To initiate LF, 50 μl of the 50-fold diluted FSB or serum sample was added to the test cassette. Cassettes were analyzed using a UCP-adapted portable reader (LFR; Qiagen, Hilden, Germany; Figure 1). The results were calculated as the ratio value (R) between T and FC signals based on relative fluorescence units (RFUs) measured at the respective lines. The cutoff for positivity (R ≥ 0.12) for the UCP-LFA batch used in this study was based on the median of a sextuple test of a standard control serum sample (+) performed in India plus the standard deviation (SD). Measurements in India yielded an identical cutoff level as in Leiden using aliquots of the same control serum sample (+). In addition, one highly anti-PGL-I IgM-positive serum sample (++) and one negative serum sample (–) were tested as control samples in sextuple to monitor the reproducibility of the test.

### Ethics

This study was performed in accordance with the Helsinki Declaration (7th revision, 64th Meeting, 2013, Fortaleza). This study was a component of Muzaffarpur NIH-TMRC HDSS for which ethics approval was received from the institutional review boards of the Institute of Medical Sciences, Banaras Hindu University (BHU; reference number Dean/2017/EC/185; Dated 24/10/2017), Varanasi, India, and the review committee of the U.S. National Institutes of Health (NIH). The institutional review board of BHU has received accreditation from the National Institutes of Health in the United States. Ethics approval was also obtained by the institutional review board of the Institute of Tropical Medicine, Antwerp (reference number 1305/19), and the Ethics Committee of the University Hospital of Antwerp (reference number 19/28/342), Belgium. All data were anonymized. Participants were informed about the study objectives, sampling protocol, and their right to refuse to take part or withdraw from the study without consequences for their treatment at any point in time. The refusal rate was lower than 5%. Written informed consent was obtained from a parent, guardian, or village head before enrollment. Consent forms were kept in a secure file cabinet at Kala-azar Medical Research Center (KAMRC), Muzaffarpur, Bihar, India.

### Statistical analysis

GraphPad Prism version 9.0.1 for Windows (GraphPad Software, San Diego, CA, USA) and RStudio version 4.2.1 (Boston, MA, USA) were used to perform statistical analysis. The distribution of anti-PGL-I data was checked for normality by plotting a histogram. Data were then log-transformed based on the natural logarithm of the anti-PGL-I R-value plus 1. Mann–Whitney U-tests and Kruskal–Wallis tests were performed to determine the statistical significance between two and three independent groups, respectively. A logistic regression analysis was performed to assess the association between age and anti-PGL-I positivity.

## Results

This was the first time applying the anti-PGL-I UCP-LFA cassette format at a larger scale in a field setting. A quality control protocol (as described in the Methods) was performed to monitor reproducibility (data not shown). The anti-PGL-I UCP-LFA was considered feasible and accepted by both healthcare staff and the population.

### Serosurvey for anti-PGL-I IgM among children in India

**Figure 2:**
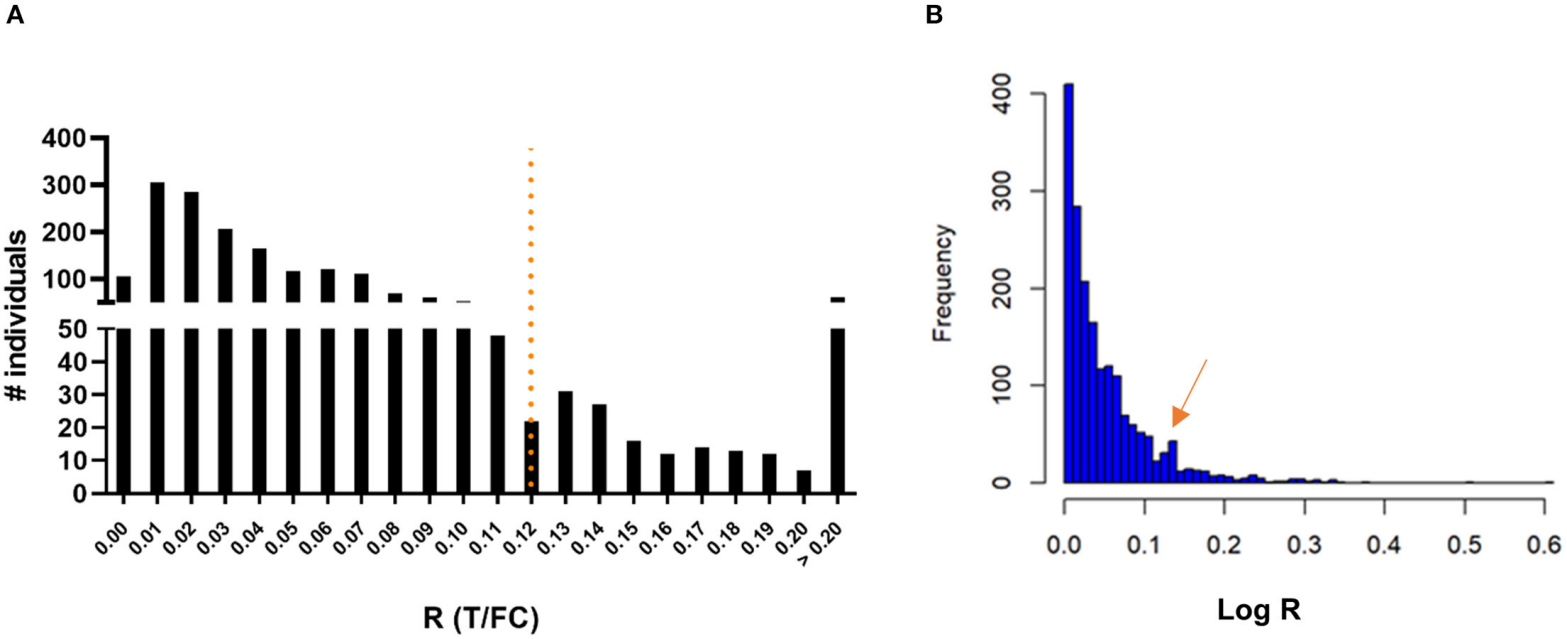
Presence of anti-PGL-I IgM antibodies in FSB of 1,857 children 3–11 years old in Bihar, India. UCP-LFA cassettes were used to obtain a quantitative R-value (T/FC) indicating the presence of anti-PGL-I IgM antibodies using a UCP reader. **(A)** Histogram with a mode of 0.01 for R: the highest R-value detected was 0.84 (*n* = 1). The cutoff for positivity (R ≥ 0.12) for the UCP-LFA batch used in this study is indicated by the dotted line and was based on the median of a sextuple test performed in India of a standard control serum sample (+) plus its standard deviation (SD). **(B)** Histogram of log-transformed R-values. FC, flow control line; IgM, immunoglobulin M; PGL-I, phenolic glycolipid I; R, ratio value, result of the UCP-LFA; T, test line.

To assess seroprevalence for anti-PGL-I IgM in a leprosy endemic area, FSB samples of 1,857 children living in the state of Bihar, India, were obtained during a field serosurvey and screened using the anti-PGL-I UCP-LFA cassette. Among these children, 215 (11.58%) tested positive for anti-PGL-I IgM (Figure 2A; Supplementary Table 1). Anti-PGL-I R values ranged from 0 to 0.84 with a median of 0.04 (IQR 0.02–0.07). The distribution appeared unimodal with a mode of 0.01 and strongly skewed to the right. Upon log transformation (based on the natural logarithm of the anti-PGL-I R-value plus 1), the distribution was still very skewed with a small dip around 0.11, followed by a much lower second mode. The log-transformed value of 0.11 corresponds with 0.12 on the original scale, and this confirms the cutoff value of R ≥ 0.12 used, as shown by the second peak in the histogram (Figure 2B). From the seropositive children with R-values >0.2 (median of seropositive children plus SD of seronegative children; *n* = 61), 51 could be followed up in October 2021 and examined for signs and symptoms of leprosy. One child (male, 9 years old) was diagnosed at follow-up at the primary healthcare center as a new paucibacillary (PB) leprosy case 1 year after the serosurvey (test performed in October 2020; R-value 0.21), showing a lesion on his left upper arm.

**Figure 3:**
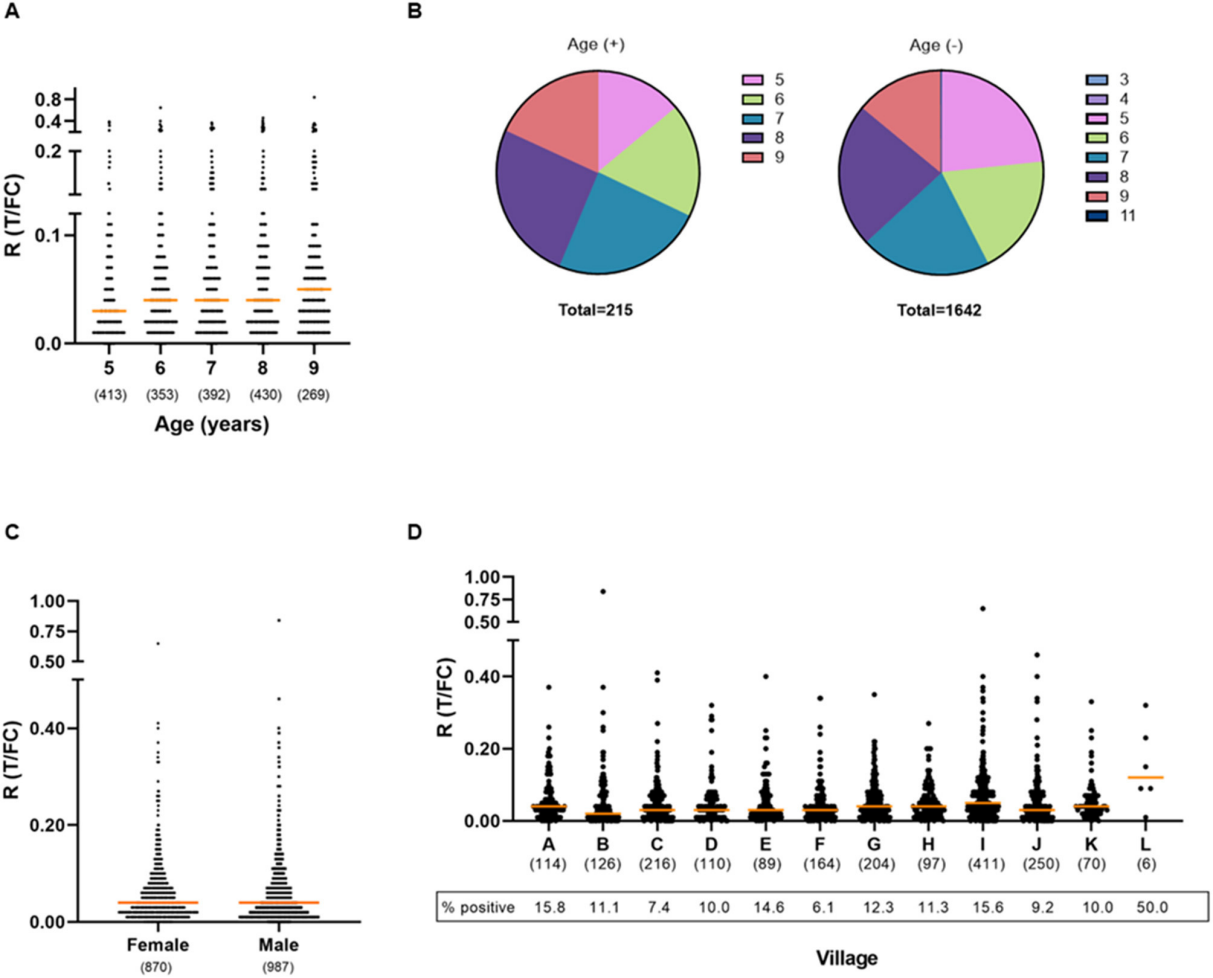
Presence of anti-PGL-I IgM antibodies in FSB of children in relation to age, gender, and place of residence. UCP-LFA cassettes were used to obtain a quantitative ratio (R) value (T/FC) indicating the presence of anti-PGL-I IgM using a UCP reader. Median values for each group are indicated by horizontal bars. The cutoff for positivity (R ≥ 0.12) for the UCP-LFA batch used in this study was based on the median of a sextuple test performed in India of a standard control serum sample (+) plus its standard deviation (SD). The numbers of individuals per group are given in parentheses. **(A)** Anti-PGL-I IgM R values per age group. Two children aged 3 years and one child aged 4 years were included in the 5-year-old age group and one child aged 11 years was included in the 9-year-old age group. **(B)** Venn diagrams displaying the proportion of children per age category for anti-PGL-I IgM-positive (+) and -negative (–) children. Colors indicate the age groups. **(C)** Anti-PGL-I IgM R values per gender. **(D)** Anti-PGL-I IgM R values per village. A: Singar Phulkahan, B: Madhopur Chhapra, C: Godai Phulkahan, D: Godai Jamal, E: Vishwanathpur, F: Raksha North, G: Raksha North Chauk, H: Raksha South West, I: Raksha South, J: Raksha Deah, K: Nariyar Nawada, L: Arizpur Kothi. FC, flow control line; IgM, immunoglobulin M; PGL-I, phenolic glycolipid I; R, ratio value, result of the UCP-LFA; T, test line.

For all participants, information on age, gender, and place of origin was recorded at the time of sampling FSB. The ages of the children in this cohort ranged from 3 to 11, with the majority of the children being 5 (*n* = 410), 6 (*n* = 353), 7 (*n* = 392), 8 (*n* = 430), or 9 (*n* = 268) years of age. For children aged 5–9 years, the percentage of seropositive children showed an increasing trend with age: from 7.32% at age 5 to 14.55% in age group 9 (Figures 3A, B; Supplementary Table 2). The odds of testing positive for anti-PGL-I IgM in this age range (5–9) increased by 18% per year (logistic regression; *p* = 0.002).

Ages were represented equally among the 987 male and 870 female participants (Table 2). No correlation was found between gender and the presence of anti-PGL-I IgM antibodies (Mann–Whitney U-test; *p* = 0.2314; Figure 3C). For female and male participants, almost identical seropositivity percentages of 11.61 and 11.55% were found, respectively.

**Table 2:** Age distribution among female and male children aged 3–11 in Bihar, India.

**Gender**	**Age**	**# Children**	**Cumulative # children**	**Percentage (%)**	**Cumulative percentage (%)**
Female	3	2	2	0.23	0.23
	4	0	2	0	0.23
	5	192	194	22.07	22.30
	6	148	342	17.01	39.31
	7	206	548	23.68	62.99
	8	203	751	23.33	86.32
	9	119	870	13.68	100
	11	0	870	0	100
Male	3	0	0	0	0
	4	1	1	0.10	0.10
	5	218	219	22.09	22.19
	6	205	424	20.77	42.96
	7	186	610	18.84	61.80
	8	227	837	23.00	84.80
	9	149	986	15.10	99.90
	11	1	987	0.10	100

The number (#) of children per age and gender are provided with the corresponding percentage of that group in relation to total male/female participants.

Children from 12 different villages were included (Supplementary Figure 1). The majority of the children were resident in Raksha South (*n* = 411). In general, seropositivity rates among the villages were similar (Figure 3D; Supplementary Table 3). However, a slight increase in levels of antibodies (R-values) among Raksha South with villages Madhopur Chhapra, Godai Phulkahan, Godai Jamal, Raksha North, and Raksha Deah was observed (Kruskal–Wallis; *p* = 0.0003 to *p* = 0.0154).

### Screening sera of leishmaniasis patients and controls

In addition to being endemic for leprosy, the area of Bihar is also endemic for leishmaniasis (32, 36). As leishmaniasis is a differential diagnosis of leprosy (37), although not the main purpose of this study, biobanked serum samples from individuals from exactly the same area with VL (*n* = 20), PKDL (*n* = 20), asymptomatic *Leishmania donovani (L. donovani)* infection (*n* = 20), and endemic controls (*n* = 20) were tested with the anti-PGL-I UCP-LFA cassette as well. Two of the 60 individuals with confirmed *L. donovani* infection tested positive (3.33%) for anti-PGL-I IgM (Table 3): one (R = 0.12) PKDL case and one person with an asymptomatic *L. donovani* infection (R = 0.26). None of the endemic controls (age 8–50) from the same area tested positive for anti-PGL-I IgM (Table 4). Moreover, plasma samples of young children (age 1–6; *n* = 37) from a non-endemic area (the Netherlands) all tested negative using the anti-PGL-I UCP-LFA cassette (Pierneef et al., *unpublished data*).

**Table 3:** Ratio values for anti-PGL-I IgM measured in individuals infected with L. donovani in Bihar, India.

**R**	**# Individuals**	**Cumulative # individuals**	**Percentage (%)**	**Cumulative percentage (%)**
0.00	15	15	25.00	25.00
0.01	16	31	26.67	51.67
0.02	12	43	20.00	71.67
0.03	7	50	11.67	83.33
0.04	2	52	3.33	86.67
0.05	2	54	3.33	90.00
0.06	2	56	3.33	93.33
0.07	1	57	1.67	95.00
0.09	1	58	1.67	96.67
0.12	1	59	1.67	98.33
0.26	1	60	1.67	100

The number (#) of individuals per ratio (R) value is provided with the corresponding percentage of that group in relation to the whole cohort. The cutoff for positivity (R ≥ 0.12) for the UCP-LFA batch used in this study was based on the median of a sextuple test performed in India of a standard control serum sample (+) plus its standard deviation (SD).

**Table 4:** Ratio values for anti-PGL-I IgM measured in endemic controls in Bihar, India.

**R**	**# Individuals**	**Cumulative # individuals**	**Percentage (%)**	**Cumulative percentage (%)**
0.00	2	2	10.00	10.00
0.01	3	5	15.00	25.00
0.02	8	13	40.00	65.00
0.03	2	15	10.00	75.00
0.04	1	16	5.00	80.00
0.05	2	18	10.00	90.00
0.09	1	19	5.00	95.00
0.10	1	20	5.00	100

The number (#) of individuals per ratio (R) value is provided with the corresponding percentage of that group in relation to the whole cohort. The cutoff for positivity (R ≥ 0.12) for the UCP-LFA batch used in this study was based on the median of a sextuple test performed in India of a standard control serum sample (+) plus its standard deviation (SD).

## Discussion

Measuring seroprevalence among young children represents a potential tool to monitor the intensity of recent transmission, particularly when antibody levels are assessed quantitatively. Changes in seroprevalence measured in repeated cross-sectional surveys among young children of a certain age group in an area can indicate the rate of transmission as well as the effect of control measures or interventions in an area. On route to leprosy elimination, an indicator for the intensity of transmission would be highly valuable, as the proportion of child cases, which is currently applied by the WHO, does not provide information swiftly as it can take years for an infected individual to develop leprosy. Moreover, it is not an accurate representation of infection as only a minority of the infected individuals develop the disease (9).

In this study, using the anti-PGL-I UCP-LFA, we found seropositivity of 11.58% among children in Bihar, a leprosy endemic state in India, with a prevalence rate of 17.1 per 10,000 population in 2019 (31), which is above the elimination threshold of 1 per 10,000 population defined by the WHO (38). In agreement with previous research, this percentage falls in line with the seroprevalence (median 14.9%) found in studies from endemic areas—mostly Brazil, India, and Indonesia (20). Seropositivity slightly increased with age (between 5 and 9 years of age). Previous reports describe a similar increase with age, followed by a decrease in antibodies after the age of 20 years (20, 39). Children of school-going age would thus be a suitable target group for sensitive assessment of recent *M*. *leprae* infection.

The slightly higher R-values (individual anti-PGL-I IgM levels) found in Raksha South compared to five other villages could not be explained by differences in the age and/or gender of the children as compared to the other villages. However, group sizes were not equal and Raksha South had a notably higher number of participants (*n* = 411), possibly affecting the analysis.

Previous studies reported that seroprevalence was stable over time if leprosy incidence in an area remained unchanged (20). A significant limitation hindering direct comparison of previous seroprevalence results from endemic areas (mostly from India, Brazil, and Indonesia) was the use of different assays measuring anti-*M. leprae*-specific antibodies. Measurements were performed using either FSB or serum with variable dilutions, and target antigens varied from native to synthetic PGL-I recognized by various IgM, IgG, or IgA isotypes. In addition, cutoff values were often chosen arbitrarily. However, analysis of data available from China showed that if the same method was used, a decrease in disease prevalence or new case detection rate corresponded to a decrease in anti-*M. leprae* antibody seroprevalence in children (21), adding the potential of measuring antibodies in children as a tool for recent transmission.

Timely diagnosis and treatment can prevent disabilities from developing and the mycobacterium from spreading (40). Active case-finding approaches by healthcare workers are a proven method to identify cases at early stages (41). In addition to the use of serology to monitor transmission, large-scale screenings have the potential to early identify new cases. Although the presence of anti-PGL-I IgM does not predict disease, seropositive individuals are at an increased risk of developing leprosy (42). In this study, 51 children were followed up and screened for clinical symptoms. One boy aged nine (anti-PGL-I IgM R-value 0.21) was diagnosed at follow-up with PB leprosy indicating the additional benefit of community seroscreening in children even for PB leprosy. As we took a cost-efficient approach, it was outside the scope of this project to follow-up all seropositive children for clinical examination which limits the additional impact of the study. In future studies, follow-up of all seropositive children as well as a subgroup of randomly selected seronegative children should be included besides additional screening of contacts of seropositive children to identify the source of transmission.

Single-dose rifampicin (SDR) PEP is a preventive treatment for leprosy which can decrease the risk of developing disease among (household) contacts of leprosy cases (43). Large-scale, international research proves that SDR-PEP is safe and the WHO recommends its use in the combat against leprosy (44). As many activities including PEP administration are ongoing worldwide and over 175,000 individuals have already received this regimen (44), it is of interest to study the effect over time on *M. leprae* transmission (measured by seroprevalence rates in children) of such prophylactic interventions and to compare the effect of PEP on transmission between countries. Crucial would be the use of one standardized, quantitative assay, which is preferentially field-friendly and easy to use at a large scale. Therefore, the low-complexity UCP-LFA cassette—which is more robust and easier to perform than the ELISA—quantitatively detecting anti-PGL-I IgM in FSB that was field-tested in this study offers a particularly suitable format for this purpose.

Since PKDL is a differential diagnosis of leprosy (37), although not the main aim of this study, we assessed the potential of the anti-PGL-I UCP-LFA to discriminate between the two infections. We found two out of 60 (3.33%) individuals infected with *L. donovani* to test positive for anti-PGL-I IgM. However, while those individuals are living in an area where both diseases are endemic, previous exposure to *M. leprae* cannot be excluded. Furthermore, in an area not endemic for leprosy, young children all tested negative in the anti-PGL-I UCP-LFA cassette, arguing for the specificity of the test. The HDSS team is experienced in conducting studies for leishmaniasis in the field, and this provides opportunities for combining leishmaniasis with leprosy research (32). Performing serosurveillance to monitor the transmission of multiple NTDs simultaneously by pooling expertise into one joint operation could save cost as well as time. To this end, the design of a (combined) rapid test for the detection of antibodies against *L. donovani* (and *M. leprae*) could also be valuable and is considered for future research (45).

## Conclusion

Screening for quantitative assessment of anti-PGL-I IgM levels in children identified 11.58% seropositivity in 12 villages in Bihar, India. These data are in line with seroprevalence data reported (20, 21) for other endemic areas without PEP in the past decades. There was a slight, significant increase with age, but no difference in seropositivity between genders was observed. Anti-PGL-I IgM antibodies in young children are a useful indicator for *M. leprae* infection, thereby serving as a proxy for recent transmission in an area and thus can be used as a tool for monitoring the reduction of transmission when new cases are scarce. A follow-up study 5 to 10 years later, again assessing the anti-PGL-I IgM antibodies in these villages in Bihar in the same age groups, would provide insight into the changes in transmission in this area. In addition, similar studies should be conducted in areas where leprosy is less endemic or not endemic anymore to further validate this tool for monitoring the elimination of transmission.

## Data availability statement

The raw data supporting the conclusions of this article will be made available by the authors, without undue reservation.

## Ethics statement

The studies involving humans were approved by Institutional Review Boards of the Institute of Medical Sciences, Banaras Hindu University (BHU; reference number Dean/2017/EC/185; Dated 24/10/2017), Varanasi, India, and the review committee of the U.S. National Institutes of Health (NIH). The studies were conducted in accordance with the local legislation and institutional requirements. Written informed consent for participation in this study was provided by the participants' legal guardians/next of kin.

## Author contributions

Conceptualization: AG. Data curation: LP, AH, MM, and ZZ. Formal analysis: LP, AH, EH, and AG. Funding acquisition: PM, SS, EH, and AG. Investigation: LP, PM, AH, SS, AS, RK, DJ, MM, AK, and ZZ. Methodology: EH and AG. Supervision: PC and AG. Visualization: LP, PM, AH, PC, EH, and AG. Writing—original draft: LP and AG. Writing—review and editing: LP, PM, AH, KC, PC, EH, and AG.

## Supplementary material

Supplementary Figure 1 Map of the research area indicating the included villages in Bihar, India, and the seropositivity percentages measured in children. The locations of 10 villages (A–J) in Bihar, India, are provided with the corresponding percentage of children testing positive for anti-PGL-I IgM. Note that two villages (K, L) were not shown on this map, as they were located too far away. UCP-LFA cassettes were used to obtain a quantitative ratio (R) value (T/FC) indicating the presence of anti-PGL-I IgM using a UCP reader. The cutoff for positivity (R ≥ 0.12) for the UCP-LFA batch used in this study was based on the median of a sextuple test performed in India of a standard control serum sample (+) plus its standard deviation (SD). A, Singar Phulkahan; B, Madhopur Chhapra; C, Godai Phulkahan; D, Godai Jamal; E, Vishwanathpur; F, Raksha North; G, Raksha North Chauk; H, Raksha South West; I, Raksha South; J, Raksha Deah; K, Nariyar Nawada; L, Arizpur Kothi. Anti-PGL-I, anti-phenolic glycolipid I; FC, flow control line; IgM, immunoglobulin M; R, ratio value, result of the UCP-LFA; T, test line.

Supplementary Table 1 Ratio values for anti-PGL-I IgM measured in children aged 3–11 in Bihar, India.

Supplementary Table 2 Anti-PGL-I IgM positivity in children aged 5–9 in Bihar.

Supplementary Table 3 Place of residence of the children aged 3–11 in Bihar and corresponding percentage of anti-PGL-I IgM positive children.
