# Supplementary figures and images for: Field-friendly anti-PGL-I serosurvey in children to monitor Mycobacterium leprae transmission in Bihar, India
Source: Front Med (Lausanne). 2023 Sep 27;10:1260375. doi: 10.3389/fmed.2023.1260375 (PMC10565223; doi:10.3389/fmed.2023.1260375)

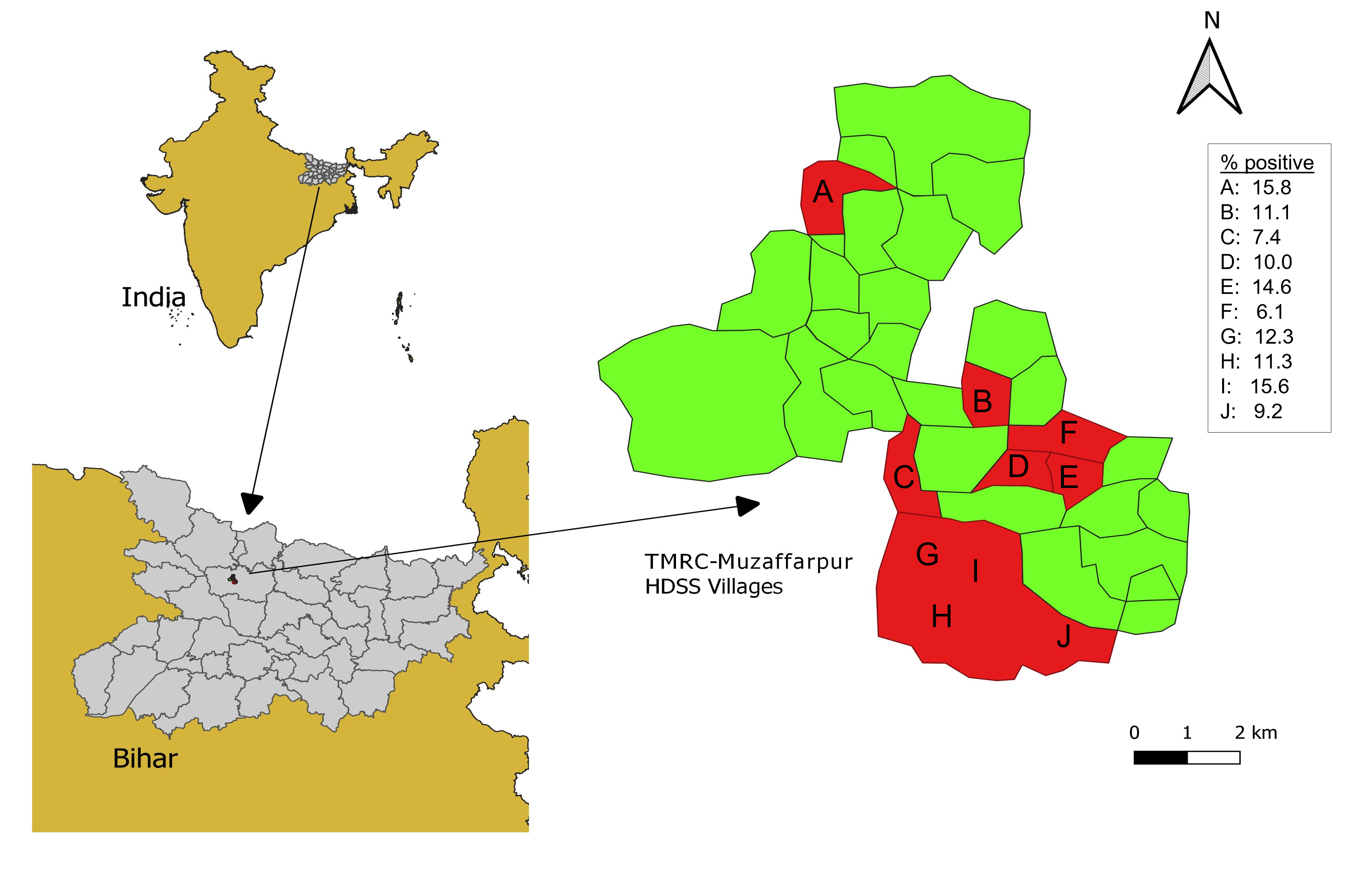

Supplement: Supplementary Figure 1 — Map of the research area indicating the included villages in Bihar, India, and the seropositivity percentages measured in children. The locations of 10 villages (A–J) in Bihar, India, are provided with the corresponding percentage of children testing positive for anti-PGL-I IgM. Note that two villages (K, L) were not shown on this map, as they were located too far away. UCP-LFA cassettes were used to obtain a quantitative ratio (R) value (T/FC) indicating the presence of anti-PGL-I IgM using a UCP reader. The cutoff for positivity (R ≥ 0.12) for the UCP-LFA batch used in this study was based on the median of a sextuple test performed in India of a standard control serum sample (+) plus its standard deviation (SD). A, Singar Phulkahan; B, Madhopur Chhapra; C, Godai Phulkahan; D, Godai Jamal; E, Vishwanathpur; F, Raksha North; G, Raksha North Chauk; H, Raksha South West; I, Raksha South; J, Raksha Deah; K, Nariyar Nawada; L, Arizpur Kothi. Anti-PGL-I, anti-phenolic glycolipid I; FC, flow control line; IgM, immunoglobulin M; R, ratio value, result of the UCP-LFA; T, test line. [file Image_1.TIF]
