# Supplementary material for: Field-friendly anti-PGL-I serosurvey in children to monitor Mycobacterium leprae transmission in Bihar, India
Source: Front Med (Lausanne). 2023 Sep 27;10:1260375. doi: 10.3389/fmed.2023.1260375 (PMC10565223; doi:10.3389/fmed.2023.1260375)
Supplement: Supplementary Table 1 — Ratio values for anti-PGL-I IgM measured in children aged 3–11 in Bihar, India. [file Table_1.docx]

**Supplemental information**

**Supplementary table 1. *Ratio values for anti-PGL-I IgM measured in children aged 3 – 11 in Bihar, India***

| R | # children | Cumulative # children | Percentage (%) | Cumulative percentage (%) |
| --- | --- | --- | --- | --- |
| 0.00 | 105 | 105 | 5.65 | 5.65 |
| 0.01 | 305 | 410 | 16.42 | 22.08 |
| 0.02 | 284 | 694 | 15.29 | 37.37 |
| 0.03 | 207 | 901 | 11.15 | 48.52 |
| 0.04 | 165 | 1,066 | 8.89 | 57.40 |
| 0.05 | 117 | 1,183 | 6.30 | 63.70 |
| 0.06 | 120 | 1,303 | 6.46 | 70.17 |
| 0.07 | 110 | 1,413 | 5.92 | 76.09 |
| 0.08 | 69 | 1,482 | 3.72 | 79.81 |
| 0.09 | 60 | 1,542 | 3.23 | 83.04 |
| 0.10 | 52 | 1,594 | 2.80 | 85.84 |
| 0.11 | 48 | 1,642 | 2.58 | 88.42 |
| 0.12 | 22 | 1,664 | 1.18 | 89.61 |
| 0.13 | 31 | 1,695 | 1.67 | 91.28 |
| 0.14 | 27 | 1,722 | 1.45 | 92.73 |
| 0.15 | 16 | 1,738 | 0.86 | 93.59 |
| 0.16 | 12 | 1,750 | 0.65 | 94.24 |
| 0.17 | 14 | 1,764 | 0.75 | 94.99 |
| 0.18 | 13 | 1,777 | 0.70 | 95.69 |
| 0.19 | 12 | 1,789 | 0.65 | 96.34 |
| 0.20 | 7 | 1,796 | 0.38 | 96.72 |
| > 0.20 | 61 | 1,857 | 3.28 | 100 |

*The number (#) of children per ratio (R) value is provided with the corresponding percentage of that group in relation to the whole cohort.* *The cut-off for positivity (R≥0.12) for the UCP-LFA batch used in this study was based on the median of a sextuple test performed in India of a standard control serum sample (+) plus its standard deviation (SD). Anti-PGL-I: anti-phenolic glycolipid I; IgM: immunoglobulin M; R: ratio value, result of the UCP-LFA.*

**Supplementary table 2. *Anti-PGL-I IgM positivity in children aged 5 – 9 in Bihar***

| Age | # children | # with R ≥0.12 (%) |
| --- | --- | --- |
| 5 | 410 | 30 (7.32) |
| 6 | 353 | 39 (11.05) |
| 7 | 392 | 52 (13.27) |
| 8 | 430 | 55 (12.79) |
| 9 | 268 | 39 (14.55) |

*For each age group the total number (#) of children, the number (percentage) testing positive for anti-PGL-I IgM are indicated. The cut-off for positivity (R≥0.12) for the UCP-LFA batch used in this study was based on the median of a sextuple test performed in India of a standard control serum sample (+) plus its standard deviation (SD). Anti-PGL-I: anti-phenolic glycolipid I; IgM: immunoglobulin M; R: ratio value, result of the UCP-LFA.*

**Supplementary table 3. *Place of residence of the children aged 3 – 11 in Bihar and corresponding percentage of anti-PGL-I IgM positive children***

| Village | Village name | # children | # with R ≥0.12 | Percentage positive per village (%) | p-value* |
| --- | --- | --- | --- | --- | --- |
| A | Singar Phulkahan | 114 | 18 | 15.79 | >0.9999 |
| B | Madhopur Chhapra | 126 | 14 | 11.11 | 0.0003 |
| C | Godai Phulkahan | 216 | 16 | 7.41 | 0.0154 |
| D | Godai Jamal | 110 | 11 | 10.00 | 0.0368 |
| E | Vishwanathpur | 89 | 13 | 14.61 | 0.6589 |
| F | Raksha North | 164 | 10 | 6.10 | 0.0011 |
| G | Raksha North Chauk | 204 | 25 | 12.25 | >0.9999 |
| H | Raksha South West | 97 | 11 | 11.34 | >0.9999 |
| I | Raksha South | 411 | 64 | 15.57 | - |
| J | Raksha Deah | 250 | 23 | 9.20 | 0.0022 |
| K | Nariyar Nawada | 70 | 7 | 10.00 | >0.9999 |
| L | Arizpur Kothi | 6 | 3 | 50.00 | >0.9999 |

*For each village the total number (#) of children, the number (percentage) testing positive for anti-PGL-I IgM are indicated. The cut-off for positivity (R≥0.12) for the UCP-LFA batch used in this study was based on the median of a sextuple test performed in India of a standard control serum sample (+) plus its standard deviation (SD). A Kruskal-Wallis test was performed to determine the statistical significance in anti-PGL-I IgM ratio (R) values between villages. The p-value* indicates the difference between the corresponding village in comparison to village Raksha South (I). Anti-PGL-I: anti-phenolic glycolipid I; IgM: immunoglobulin M; R: ratio value, result of the UCP-LFA.*

This is a provisional file, not the final typeset article
